# Supplementary material for: Multi‐dataset identification of innovative feature genes and molecular mechanisms in keratoconus
Source: J Cell Mol Med. 2024 Sep 19;28(18):e70079. doi: 10.1111/jcmm.70079 (PMC11412914; doi:10.1111/jcmm.70079)
Supplement: Supplementary file 2 — Material S2. [file JCMM-28-e70079-s002.zip › 7.GSEA analysis/5.RNF217-AS1/gsea_report_for_h_1678852909625.html]

Report for h 1678852909625 [GSEA]

| GS  follow link to MSigDB | GS DETAILS | SIZE | ES | NES | NOM p-val | FDR q-val | FWER p-val | RANK AT MAX | LEADING EDGE || 1 | KEGG\_PHENYLALANINE\_METABOLISM | Details ... | 17 | 0.53 | 1.48 | 0.021 | 1.000 | 0.808 | 5673 | tags=29%, list=16%, signal=35% |
| 2 | KEGG\_RNA\_DEGRADATION | Details ... | 51 | 0.22 | 1.15 | 0.246 | 1.000 | 0.993 | 26981 | tags=100%, list=78%, signal=448% |
| 3 | KEGG\_RIBOSOME | Details ... | 85 | 0.29 | 1.06 | 0.345 | 1.000 | 0.998 | 21326 | tags=98%, list=61%, signal=252% |
| 4 | KEGG\_PENTOSE\_AND\_GLUCURONATE\_INTERCONVERSIONS | Details ... | 20 | 0.40 | 1.06 | 0.376 | 1.000 | 0.998 | 10697 | tags=45%, list=31%, signal=65% |
| 5 | KEGG\_TERPENOID\_BACKBONE\_BIOSYNTHESIS | Details ... | 15 | 0.41 | 1.05 | 0.441 | 1.000 | 0.998 | 568 | tags=7%, list=2%, signal=7% |
| 6 | KEGG\_REGULATION\_OF\_AUTOPHAGY | Details ... | 30 | 0.33 | 1.01 | 0.425 | 1.000 | 0.999 | 2619 | tags=13%, list=8%, signal=14% |
| 7 | KEGG\_CYSTEINE\_AND\_METHIONINE\_METABOLISM | Details ... | 34 | 0.22 | 0.97 | 0.494 | 1.000 | 0.999 | 1456 | tags=6%, list=4%, signal=6% |
| 8 | KEGG\_GLYCEROLIPID\_METABOLISM | Details ... | 43 | 0.27 | 0.93 | 0.584 | 1.000 | 1.000 | 6786 | tags=33%, list=20%, signal=40% |
| 9 | KEGG\_B\_CELL\_RECEPTOR\_SIGNALING\_PATHWAY | Details ... | 75 | 0.23 | 0.92 | 0.602 | 1.000 | 1.000 | 1532 | tags=8%, list=4%, signal=8% |
| 10 | KEGG\_NEUROACTIVE\_LIGAND\_RECEPTOR\_INTERACTION | Details ... | 270 | 0.25 | 0.90 | 0.786 | 1.000 | 1.000 | 8109 | tags=30%, list=23%, signal=39% |
| 11 | KEGG\_PORPHYRIN\_AND\_CHLOROPHYLL\_METABOLISM | Details ... | 32 | 0.27 | 0.88 | 0.656 | 1.000 | 1.000 | 7285 | tags=22%, list=21%, signal=28% |
| 12 | KEGG\_GNRH\_SIGNALING\_PATHWAY | Details ... | 100 | 0.19 | 0.86 | 0.716 | 1.000 | 1.000 | 9095 | tags=24%, list=26%, signal=32% |
| 13 | KEGG\_AUTOIMMUNE\_THYROID\_DISEASE | Details ... | 48 | 0.31 | 0.86 | 0.663 | 1.000 | 1.000 | 3819 | tags=19%, list=11%, signal=21% |
| 14 | KEGG\_STEROID\_BIOSYNTHESIS | Details ... | 17 | 0.28 | 0.86 | 0.624 | 1.000 | 1.000 | 11271 | tags=35%, list=32%, signal=52% |
| 15 | KEGG\_PROTEASOME | Details ... | 43 | 0.24 | 0.85 | 0.665 | 1.000 | 1.000 | 474 | tags=2%, list=1%, signal=2% |
| 16 | KEGG\_GLYOXYLATE\_AND\_DICARBOXYLATE\_METABOLISM | Details ... | 16 | 0.30 | 0.85 | 0.639 | 1.000 | 1.000 | 729 | tags=6%, list=2%, signal=6% |
| 17 | KEGG\_PENTOSE\_PHOSPHATE\_PATHWAY | Details ... | 27 | 0.22 | 0.84 | 0.747 | 1.000 | 1.000 | 280 | tags=4%, list=1%, signal=4% |
| 18 | KEGG\_PROTEIN\_EXPORT | Details ... | 22 | 0.26 | 0.83 | 0.742 | 1.000 | 1.000 | 1167 | tags=5%, list=3%, signal=5% |
| 19 | KEGG\_OLFACTORY\_TRANSDUCTION | Details ... | 372 | 0.25 | 0.83 | 0.955 | 1.000 | 1.000 | 6565 | tags=27%, list=19%, signal=33% |
| 20 | KEGG\_VASCULAR\_SMOOTH\_MUSCLE\_CONTRACTION | Details ... | 111 | 0.22 | 0.77 | 0.834 | 1.000 | 1.000 | 7735 | tags=24%, list=22%, signal=31% |
| 21 | KEGG\_MATURITY\_ONSET\_DIABETES\_OF\_THE\_YOUNG | Details ... | 25 | 0.29 | 0.77 | 0.838 | 1.000 | 1.000 | 2984 | tags=16%, list=9%, signal=17% |
| 22 | KEGG\_GAP\_JUNCTION | Details ... | 86 | 0.17 | 0.76 | 0.947 | 1.000 | 1.000 | 7550 | tags=19%, list=22%, signal=24% |
| 23 | KEGG\_GLYCINE\_SERINE\_AND\_THREONINE\_METABOLISM | Details ... | 31 | 0.22 | 0.75 | 0.944 | 1.000 | 1.000 | 5787 | tags=19%, list=17%, signal=23% |
| 24 | KEGG\_ASCORBATE\_AND\_ALDARATE\_METABOLISM | Details ... | 17 | 0.28 | 0.74 | 0.837 | 1.000 | 1.000 | 7074 | tags=29%, list=20%, signal=37% |
| 25 | KEGG\_NATURAL\_KILLER\_CELL\_MEDIATED\_CYTOTOXICITY | Details ... | 125 | 0.20 | 0.74 | 0.953 | 1.000 | 1.000 | 2619 | tags=9%, list=8%, signal=9% |
| 26 | KEGG\_OOCYTE\_MEIOSIS | Details ... | 111 | 0.14 | 0.74 | 0.809 | 1.000 | 1.000 | 7161 | tags=13%, list=21%, signal=16% |
| 27 | KEGG\_NOTCH\_SIGNALING\_PATHWAY | Details ... | 47 | 0.19 | 0.72 | 0.780 | 1.000 | 1.000 | 1227 | tags=4%, list=4%, signal=4% |
| 28 | KEGG\_RENIN\_ANGIOTENSIN\_SYSTEM | Details ... | 17 | 0.32 | 0.72 | 0.862 | 1.000 | 1.000 | 5655 | tags=24%, list=16%, signal=28% |
| 29 | KEGG\_RIG\_I\_LIKE\_RECEPTOR\_SIGNALING\_PATHWAY | Details ... | 64 | 0.19 | 0.71 | 0.909 | 0.993 | 1.000 | 2619 | tags=8%, list=8%, signal=8% |
| 30 | KEGG\_DRUG\_METABOLISM\_OTHER\_ENZYMES | Details ... | 42 | 0.23 | 0.70 | 0.937 | 0.962 | 1.000 | 7074 | tags=21%, list=20%, signal=27% |
| 31 | KEGG\_GLYCOSAMINOGLYCAN\_DEGRADATION | Details ... | 21 | 0.22 | 0.67 | 0.857 | 0.973 | 1.000 | 4106 | tags=10%, list=12%, signal=11% |
| 32 | KEGG\_MTOR\_SIGNALING\_PATHWAY | Details ... | 51 | 0.19 | 0.65 | 0.849 | 0.960 | 1.000 | 7112 | tags=16%, list=20%, signal=20% |
| 33 | KEGG\_METABOLISM\_OF\_XENOBIOTICS\_BY\_CYTOCHROME\_P450 | Details ... | 60 | 0.18 | 0.57 | 1.000 | 0.993 | 1.000 | 7908 | tags=25%, list=23%, signal=32% |
| 34 | KEGG\_BIOSYNTHESIS\_OF\_UNSATURATED\_FATTY\_ACIDS | Details ... | 19 | 0.16 | 0.57 | 0.980 | 0.964 | 1.000 | 9226 | tags=21%, list=27%, signal=29% |
Table: Gene sets enriched in phenotype **h (8 samples) **[plain text format]****
